# *Bacillus subtilis* DSM29784 Alleviates Negative Effects on Growth Performance in Broilers by Improving the Intestinal Health Under Necrotic Enteritis Challenge

**DOI:** 10.3389/fmicb.2021.723187

**Published:** 2021-09-16

**Authors:** Yuanyuan Wang, Yibin Xu, Shengliang Xu, Jinyong Yang, Kaiying Wang, Xiuan Zhan

**Affiliations:** ^1^Institute of Feed Science, College of Animal Sciences, Zhejiang University, Hangzhou, China; ^2^Haiyan Animal Husbandry and Veterinary Bureau, Haiyan, China; ^3^Zhejiang Animal Husbandry Technology Extension and Livestock and Poultry Monitoring Station, Hangzhou, China

**Keywords:** performance, broilers, subclinical necrotic enteritis, *Bacillus subtilis* DSM29784, microbiota, metabolome

## Abstract

Along with banning antibiotics, necrotic enteritis (NE), especially subclinical enteritis (SNE), poses a significant threat to the chicken industry; however, probiotics are a potentially promising intervention. We aimed to investigate the beneficial effects of *Bacillus subtilis* DSM29784 (BS) on the treatment of *Clostridium perfringens* (CP)-induced SNE in broilers. A total of 360 1-day-old broiler chicks were divided into three treatment groups, namely control (Ctr), SNE, and BS treatment (BST) groups, all of which were fed with a basal died for 21days, and then from day 22 onward, only the BST group had a BS supplemented diet (1×10^9^ colony-forming units BS/kg). On day 15, all chicks, except the Ctr group, were challenged with a 20-fold dose coccidiosis vaccine and 1ml CP (2×10^8^) on days 18–21 for SNE induction. Beneficial effects were observed on growth performance in BST compared to SNE broilers. BST treatment alleviated intestinal lesions and increased the villus height/crypt depth ratio. Further, BST broilers showed increased maltase activity in the duodenum compared with SNE chicks, and a significantly decreased caspase-3 protein expression in the jejunum mucosa. Moreover, an increased abundance of *Ruminococcaceae* and *Bifidobacterium* beneficial gut bacteria and an altered gut metabolome were observed. Taken together, we demonstrate that the manipulation of microbial gut composition using probiotics may be a promising prevention strategy for SNE by improving the composition and metabolism of the intestinal microbiota, intestinal structure, and reducing inflammation and apoptosis. Hence, BS potentially has active ingredients that may be used as antibiotic substitutes and effectively reduces the economic losses caused by SNE. The findings of this study provide a scientific foundation for BS application in broiler feed in the future.

## Introduction

Necrotic enteritis (NE) is an enteric disease with a significant economic burden, in terms of mortality and welfare, on the poultry industry, especially in broiler chickens in China (Wu et al., 2012; Gu et al., 2019; Khan et al., 2021). The causative agent of NE is *Clostridium perfringens* (CP), a ubiquitous Gram-positive bacterium that produces spores and highly potent toxins (Gohari et al., 2021). Clinical NE is characterized by high mortality in poultry, while subclinical NE (SNE), which is becoming more prevalent, is mainly characterized by intestinal mucosal damage without clinical signs or mortality (Rajput et al., 2020). In addition, SNE causes wet litter (Williams, 2005) and possible contamination of poultry products for human consumption (Villagran-de la Mora et al., 2020). Gastrointestinal tract infections are believed to cause annual losses of 2–6 billion USD to the global poultry industry (Dahiya et al., 2006). However, microbial-based therapy improved the economic benefit of broilers with NE (Sallam et al., 2021).

Antibiotics are commonly used as growth promoters and for prophylaxis of SNE (Prescott et al., 2016). Residues of these antibiotics in poultry products have harmful effects on human health, such as antibiotic resistance (Bani-Asadi et al., 2021). However, antibiotics have been banned as feed additives in China and many other regions; in the absence of antibiotics alternatives, chickens raised under current intensive production systems face a higher risk of enteric pathogen infection (Lee et al., 2015). Clostridial diseases in poultry, such as NE, have once again become a disease of worldwide economic importance, and hence identifying antibiotic alternatives for disease control in poultry remains essential.

Currently, probiotics are the most extensively used alternatives to enhance growth and improve health in poultry production (Hofacre et al., 2018; Rodrigues et al., 2018). Probiotics are live microbial supplements that confer health benefits to the host when administered in adequate amounts (Alagawany et al., 2018). Studies have shown that probiotics favor intestinal health and the production performance of broilers by exerting beneficial effects on intestinal morphology, microflora, nutrient absorption, antioxidative capacity, and immune response (Wang et al., 2017b; Chen et al., 2018; Rodjan et al., 2018; Wu et al., 2019). Therefore, management of poultry gut health is an important strategy for maintaining productivity in the post-antibiotic era. The use of probiotics is one such strategy that has gained attention over recent years (Shini and Bryden, 2021) with accumulating evidence showing that probiotics have a significant effect in preventing NE (Caly et al., 2015; Li et al., 2017b; Khalique et al., 2020; Zhao et al., 2020). Among the various bacterial species used as probiotics, *Bacillus* spp. strains are the most promising feed supplements for poultry due to their spores exhibiting health-promoting benefits, as well as having the capacity to survive harsh environmental conditions, such as the high temperatures used in the pelleting process and the low pH in the gastrointestinal tract (Shivaramaiah et al., 2011). *Bacillus* spores improve gut health by competitive exclusion, production of antimicrobial peptides and beneficial metabolites, and stimulation of the intestinal immune system (Hayashi et al., 2018). *B. subtilis*, which has a broad activity against Clostridium spp., and improves overall performance in broilers (Jayaraman et al., 2013).

The interface of the immune system and metabolism is an emerging field of research (Liu et al., 2021). Metabolomics is an effective method for describing the global metabolism of living organisms and capturing the metabolic changes associated with external stimuli (Zhang et al., 2017). Generally, host and their intestinal microorganisms form the host–microbial–metabolic axis, whereas the normal microflora in the gut can metabolize ingested and endogenous macromolecular carbohydrates such as proteins and fatty acids. In addition, microorganisms interact with the metabolism of organisms to produce various metabolites such as short-chain fatty acids, amino acids, and peptides. These metabolites play a vital role in the immune homeostasis of intestinal tissues, and even the whole body (Li et al., 2011; Duca et al., 2014). Therefore, an integrated immuno-metabolic approach can identify potential therapeutic targets. Previous studies have demonstrated that *B. subtilis* DSM29784 (BS) can improve the growth performance and gut health in turkeys (Mohammadigheisar et al., 2019). We have previously reported that supplementary probiotic BS could be used as an alternative to antibiotics for broiler chickens, by decreasing the feed conversion rate and improving intestinal health (Wang et al., 2021). However, reports on the effects of BS supplementation on the treatment of SNE broilers are scarce. Therefore, the aims of this study were to investigate the effects of BS supplementation as a treatment on the growth performance and the composition, and metabolism, of the intestinal microbiota, intestinal structure of broiler chickens following CP challenge, and determine whether BS supplementation reduces poor growth performance caused by CP in broiler chickens.

## Materials and Methods

All procedures herein were conducted in accordance with the Chinese Guidelines for Animal Welfare and approved by the Zhejiang University Institutional Animal Care and Use Committee (Permission number: ZJU2019-480-12).

### Probiotic Bacterial Culture and Powder Preparation

The probiotic *B. subtilis* DSM29784 strain (BS) used in this study was presented by the Chinese Academy of Sciences (Beijing, China). Bacteria were cultured overnight in Luria–Bertani broth (Solarbio, No. L1010, Beijing, China) at 180rpm in a shaking incubator (Jiecheng, Shanghai, China) at 37°C and were then pelleted by centrifugation at 5,000×*g* for 10min at 4°C. The pellets were then washed twice with sterile phosphate buffer saline (PBS; pH 7.3; Aladdin, Shanghai, China), resuspended in skim milk powder (Devondale, Australia), and diluted to the final concentration of 2×10^9^cfu/g.

### Experimental Design and Bird Husbandry

A total of 360 1-day-old Lingnan Yellow broiler chicks with similar body weight were purchased from Xingjian hatchery (Jiaxing, China). Chicks were weighted and divided into 3 treatment groups. Each group consisted of 6 replicates with 20 birds (10 males and 10 females) per replicate with a total of 18 floor pens (area of 2m×4m) covered with fresh wood shavings. The temperature was maintained at 32–34°C for the first 3days and then reduced by 2–3°C per week, to a final temperature of 26°C with a humidity of 60–65%. Birds were given *ad libitum* access to water and food throughout the experiment (63days) and were kept under full light for the first 3days, and then changed to light–dark (2 Light–Dark) cycle. Chicks were fed with diets in pellet form as follows: negative control group (Ctr), positive control group (SNE), and BS treatment group (BST) fed with a basal died for 21days and then, from day 22 onward, only the BST group had a BS (1×10^9^ colony-forming units BS/kg) supplemented diet. The composition and nutrient concentration of the basal diet are listed in Table 1.

**Table 1:** Composition and nutrient level of the basal diet used in different phases of trial (% as fed basis).

Ingredients, %	Starter (1–21days)	Grower (22–42days)	Finisher (43–63days)
Corn	62.50	67.50	75.00
Soybean meal	31.00	23.50	14.50
CPM^c^	2.00	4.00	5.00
Soybean oil	0.50	1.00	1.50
NaCl	0.30	0.30	0.30
CaHPO_4_	1.20	1.00	0.80
Limestone	1.50	1.30	1.20
Zeolite	-	0.40	0.70
Premix^a^	1.00	1.00	1.00
Total	100.00	100.00	100.00
Nutrient levels^b^ (%)			
ME (MJ/kg)	12.22	12.59	12.97
CP	21.09	19.16	16.07
Lys	1.09	0.99	0.87
Met	0.49	0.38	0.35
Met+Cys	0.87	0.73	0.65
Calcium	0.90	0.85	0.69
Total phosphorus	0.58	0.52	0.45

a The Premix provides per kg of diet: Fe 80mg, Cu 8mg, Zn 60mg, Mn 80mg, I 0.35mg, Se 0.15mg, V_A_ 9,600IU, V_D3_ 1,500IU, V_E_ 20mg, V_k3_ 1mg, V_B1_ 2.2mg, V_B2_ 4.2mg, V_B6_ 4.2mg, V_B12_ 0.012mg, nicotinamide 42mg, D-calcium pantothenate 12mg, folic acid 1.0mg, D-biotin 0.18mg, and choline 800mg.

b ME is a calculated value; other nutrient levels are measured values.

c CPM is corn gluten meal.

### *Clostridium perfringens* Culture and Coccidiosis Vaccine

The *C. perfringens* type-A strain (American Type Culture Collection 13124) was obtained from the Guangdong Microbial Culture Collection Center (Guangzhou, China). The strain was aseptically inoculated into Reinforced Clostridial Medium (Guangdong HuanKai Microbial Sci. & Tech. Co., Ltd., Guangdong, China) overnight at 37°C in an anaerobic environment before being used for the challenge. The live coccidiosis quadrivalent vaccine for chickens (Eimeria tenella strain PTMZ, E. necatrix strain PNHZ, E. maxima strain PMHY, and E. acervulina strain PAHY) was provided by Foshan Zhengdian Biotechnology Co., Ltd. (Guangdong, China).

### Subclinical Necrotic Enteritis Broiler Model

SNE was induced in broilers as previously described (Wu et al., 2018) with minor modifications. Briefly, prior to SNE challenge (day 14), birds were given sufficient water, but no food was provided overnight. On day 15, the SNE-challenged and BST groups received 20 times more concentrated than usual per bird by oral gavage. The birds in each group were then gavaged with 1ml of *C. perfringens* (2×10^8^cfu/ml) on days 18–21, and the food was cut off overnight but water was provided before each challenge. Meanwhile, the birds in the Ctr group received equivalent sterile PBS instead at day 15, and 1ml of sterile Reinforced Clostridium Medium (Guangdong HuanKai Microbial Sci. &Tech. Co., Ltd.) was administered daily on days 18–21. Samples were harvested on days 28 and 35.

### Measurement of Growth Performance and Harvesting Samples

Feed intake and body weight (BW) of broilers was measured per replicate as the unit on days 1, 21, 42, and 63. Mortality was monitored daily; dead birds were recorded and weighed to adjust the estimates of gain, intake, and feed conversion ratios as appropriate. The average daily gain, average daily feed intake (ADFI), and feed: gain ratio (F:G) were calculated.

On days 28 and 35, 12 birds (2 birds per replicate) from each group were randomly selected and weighed after withdrawing feed, but still providing fresh water, for 12h. The chicks were electrically stunned, exsanguinated, and dissected by a trained team who harvested tissue samples. First, the small intestine from each bird was removed, opened, and scored for intestinal lesions, by the same trained observer, using the following scale: 0, no gross lesions; 1, thin, friable small intestine; 2, focal necrosis, ulceration, or both; 3, patchy necrosis; and 4, severe, extensive mucosal necrosis (Johnson and Reid, 1970). The average score was computed per pen, and the pen was the experimental unit for lesion scoring. Following this, 0.5cm of the upper jejunum wall was fixed in 25% glutaraldehyde (pH 74; Aladdin, Shanghai, China) and 4% paraformaldehyde (Aladdin, Shanghai, China), respectively, and the mucosa of the remaining jejunum, and the middle segments of the duodenum were gently scraped. The upper portion of the cecum was then tied with a string and snap-frozen in liquid nitrogen. In addition, the contents of the cecum were gently scraped with a blade and stored at – 80°C until analysis.

Duodenal and jejunal mucosa samples (1g) were homogenized in 9ml of 0.9% sterile saline (Aladdin, Shanghai, China) on ice and centrifuged at 3,000×*g* for 15min at 4°C. The total protein concentration of the supernatant was measured using a Pierce™ bicinchoninic acid (BCA, No. A045-4-2) Protein Assay Kit, according to the manufacturer’s protocol (Thermo Fisher Scientific). The prepared supernatant was stored at – 80°C and used for test.

### Jejunum Morphology

At necropsy, jejunal tissue samples were harvested and fixed in 4% paraformaldehyde (No. BL539A, Biosharp, Beijing, China), dehydrated, and processed into paraffin sections according to a standard procedure (Wu et al., 2019). Paraffin sections were then subjected to hematoxylin and eosin staining (H&E; Google Bio, Wuhan, China) staining for histopathological analysis (Xu et al., 2018) Transmission electron microscopy (Wang et al., 2018) and scanning electron microscopy (SEM; Hu et al., 2018) of the jejunal tissue were conducted according to previous studies.

### Histomorphological Measurements of the Jejunum

Morphometric measurements of jejunal villi were performed at 40× magnification using a light microscope (Precise, Beijing, China). The criterion for villus selection was based on the presence of an intact lamina propria. Villi length was measured from the tip of the villus to the villus-crypt junction; whereas crypt depth was defined as the depth of the invagination between adjacent villi (Awad et al., 2009).

### Total RNA Extraction and Quantitative Real-Time PCR

Based on a previously described methods (Wang et al., 2019), RNA was extracted from the intestinal mucosa using RNAiso Plus reagent (No. RR047A, TaKaRa Bio, Kusatsu, Japan) according to the manufacturer’s instructions. Complementary DNA (cDNA) was synthesized from 1μg of total RNA using M-MLV reverse transcriptase (No. RR420A, TaKaRa Bio) according to the manufacturer’s instructions. Transcriptional changes were then identified by quantitative PCR (qPCR), using SYBR® Green Premix Ex Taq™ (TaKaRa) and the ABI 7500 Fast Real-Time PCR system (Applied Biosystems, Carlsbad, CA, United States). Thermocycling was as follows: 95°C for 30s, followed by 40cycles of 95°C for 5s and 60°C for 34s, and finally a melting curve analysis to monitor the purity of the PCR product. Primer sequences are shown in Table 2. The 2^−ΔΔCT^ method was used to estimate mRNA abundance. ΔCt is Ct, target−Ct, reference, and ΔΔCt is ΔCt, treatment−ΔCt, control. Relative gene expression levels were normalized to those of the eukaryotic reference gene, β-actin.

**Table 2:** Sequences of real-time PCR primers.

Gene name	Primers (5'-3')	Products	GenBank
*Claudin-1*	F: TGGCCACGTCATGGTATGG	62	NM_001013611
	R: AACGGGTGTGAAAGGGTCATAG		
*Occluding*	F: GAGCCCAGACTACCAAAGCAA	68	NM_205128
	R: GCTTGATGTGGAAGAGCTTGTTG		
*Muc-2*	F: GCCTGCCCAGGAAATCAAG	59	NM_001318434
	R: CGACAAGTTTGCTGGCACAT		
*β -actin*	F: GAGAAATTGTGCGTGACATCA	152	NM_205518
	R: CCTGAACCTCTCATTGCCA		

Muc-2, Mucin-2.

### Biochemical Determinations

The enzymatic activities of sucrase (No. A082-2-1), amylase (No. C016-1-1), and maltase (No. A082-3-1) in the duodenal mucosa were determined using colorimetric methods and measured with a spectrophotometer (BioMate 5; Thermo Electron Corporation, Hemel Hempstead, United Kingdom). The assays were conducted using assay kits according to the manufacturer’s instructions (Nanjing Jiancheng Bioengineering Institute, Nanjing, China). Absorbance was measured using an Infinite M200 Pro NanoQuant™ (Tecan, Mannedorf, Switzerland).

### ELISA Determinations

Levels of secretory immunoglobulin A (sIgA, No. H108-2), immunoglobulin G (IgG, No. H106), interleukin (IL)-6 (No. H007-1-2), IL-1β (No. H002), tumor necrosis factor α (TNF-α, No. H052-1), interferon-gamma (IFN-γ, No. H025), Bcl-2 (No. H073), Bax (No. H379-1), and caspase-3 (No. H076) in the jejunal mucosa were determined by ELISA (Nanjing Jiancheng Institute of Bioengineering) according to the manufacturer’s instructions. Briefly, supernatant from jejunal mucosal samples were pipetted into enzyme wells, which has been pre-coated with antibodies specific for sIgA, IgG, IL-1β, TNF-α, IFN-γ, IL-6, Bcl-2, Bax, and caspase-3, then add recognition antigen labeled by horse radish peroxidase (HRP); after been incubated 30min at 37°C, both compete with solid phase antigen and formed immune complex; after been washing by Phosphate Buffered Saline Tween (PBST), the combined HRP catalyzes Tetramethy1 benzidine (TMB) into blue, and turns into yellow by the action of acid; it has absorption peak under 540nm wavelength, and the absorbance of each well was determined using a SpectraMax M5 (Molecular Devices, San Jose, CA, United States; Li et al., 2017a).

### Cecal DNA Extraction and 16S rRNA Gene Sequencing

Microbial genomic DNA was extracted from freeze-dried cecal content samples using a commercial magnetic bead DNA isolation kit purchased from Hangzhou Foreal Nanotechnology (Hangzhou, China). The V3–V4 region of the bacterial 16S rRNA gene was amplified using the 341F/806R primer pair combined with adapter and barcode sequences. PCR products were quantified using Quant-iT dsDNA HS Reagent (Thermo Fisher Scientific, Suzhou, China) and pooled. High-throughput sequencing analysis of bacterial rRNA genes was performed using the Illumina HiSeq 2,500 platform (2×250 paired ends; Illumina, San Diego, CA, United States) at Biomarker Technologies Corporation (Beijing, China). Raw high-throughput sequence data were deposited in the NCBI database with the BioProject ID PRJNA713493.^1^

The QIIME (version 1.9.1) data analysis package was used for 16S rRNA data analysis.^2^ The original sequence data were spliced using FLASH (version 1.2.11; Magoc and Salzberg, 2011) and then subjected to mass filtering with Trimmomatic (version 0.33; Bolger et al., 2014). The effective sequences were grouped into operational taxonomic units (OTUs) using the clustering program USEARCH (version 10.0; Edgar, 2013), against the Silva 119 database, preclustered at 97% sequence identity. The OTUs were further subjected to taxonomy-based analysis using the Remove Data Processor algorithm, and α diversity and β diversity were analyzed. Linear discriminant analysis, Effect Size (LEfSe; version 1.0) analysis, and linear discriminant analysis (LDA) were performed using an online LEfSe tool.^3^

### Measurement of Cecal Metabolites

Based on a previously described method (Sun et al., 2020), frozen cecal digesta (0.5g) were lyophilized for 24h and transferred into a 1-ml polyethylene tube. The digesta was combined with 100μl of methoxyamine hydrochloride in pyridine (20mg/ml) and vigorously vortexed for 30s. The samples were heated in a water bath at 37°C for 90min, followed by the addition of 200μl of bis (trimethylsilyl)-trifluoroacetamide in 1% trimethylchlorosilane. The samples were then heated at 70°C for 60min, removed, and allowed to remain at room temperature for 30min. Subsequently, the samples were centrifuged at 10,000×g at 4°C for 10min, and 100μl of the supernatant from each sample was transferred into a Gas Chromatography vial. Following the addition of 400–500μl of n-hexane, the samples were used for Gas Chromatography–Mass Spectrometry (GC–MS) in the automatic sampling mode.

Each 1μl sample was injected into the Agilent 6890/5973 system equipped with a fused silica capillary column (30.0m×0.25mm i.d.) packed with 0.25μm HP-5MS (Agilent, Santa Clare, CA, United States). Helium was used as the carrier at a constant flow rate of 1.0ml/min. The column temperature was maintained at 70°C for 2min, increased to 200°C at a rate of 10°C/min, followed by an increase to 280°C at a rate of 5°C/min, which was maintained for 6min. The full scanning mode was adopted for mass spectrometry detection with a detection range of 50–650 (m/Z).

### Statistical Analysis

The metabolic profile data were processed using Soft Independent Modeling of Class Analogy software (version 13.0; Sartorius Stedim Data Analytics AB, Umea, Sweden). Principal component analysis (PCA), projections to latent structures-discriminant analysis (PLS-DA), and orthogonal partial least squares discriminant analysis (OPLS-DA) were used to process the cecum metabolomic data. Variable importance in projection (VIP>1) and Welch’s *t*-test (*p*<0.05) values were compared to obtain the profile of each metabolite. Kyoto Encyclopedia of Genes and Genomes (KEGG) enrichment analysis was used, and all qualitative metabolites were taken as the background. The Fisher’s exact test was used to analyze and calculate the degree of enrichment of the metabolites in each pathway. Spearman correlation analysis was performed for cecal metabolites and microbiota.

Other data were analyzed with a one-way ANOVA followed by Tukey’s multiple comparison test using Statistical Product and Service Solutions (SPSS) software (version 22.0; SPSS Inc., Chicago, IL, United States). Statistical significance was set at *p*<0.05. The data were expressed as mean±SEM, and graphs were generated using GraphPad Prism software (version 5.0; San Diego, CA, United States).

## Results

### Growth Performance of Broilers

Growth performance results are presented in Table 3. During the starter phase from days 1–42, there was no significant effect on BW, Average Daily Gain (ADG), ADFI, and F:G because of BS supplementation when compared with SNE group (*p*>0.05). In contrast, from days 43–63, treatment with BS improved the BW of broilers. Moreover, considering the entire growth period from days 1 to 63, the ADG of broilers in the BST group was enhanced when compared with the SNE group (*p*<0.05). Furthermore, the SNE group had a lower BW in the final phase from days 43 to 63 and lower ADG during the whole experiment than the other two groups (*p*<0.05). However, the Ctr group showed significantly higher BW and ADG, and lower F/G in each period compared to the SNE group (*p*<0.05).

**Table 3:** Effects of *Bacillus subtilis* DSM29784 treatment group on the growth performance of subclinical enteritis (SNE) broilers.

Items	Ctr	SNE	*Bacillus subtilis* treatment (BST)	SEM	*p*
Starter phase (days 1–21)					
BW at day 21 (g)	555.80^a^	528.97^b^	525.33^b^	11.76	0.040
ADG (g/d)	24.34^a^	23.06^b^	22.87^b^	0.567	0.042
ADFI (g/d)	42.04	42.53	41.89	0.954	0.785
F:G	1.73^b^	1.85^a^	1.83^a^	0.029	0.002
Grower phase (days 22~42d)					
BW at day 42 (g)	1615.01^a^	1374.69^b^	1407.67^b^	32.885	<0.001
ADG (g/d)	50.44^a^	40.27^b^	42.02^b^	1.513	<0.001
ADFI (g/d)	122.15	111.41	109.37	6.069	0.111
F:G	2.42^b^	2.76^a^	2.61^ab^	0.102	0.017
Finisher phase (days 43~63d)					
BW at day 63 (g)	2683.55^a^	2221.81^c^	2312.58^b^	36.193	<0.001
ADG (g/d)	50.88^a^	40.34^b^	43.09^b^	1.398	<0.001
ADFI (g/d)	153.74	140.78	144.33	7.045	0.198
F:G	3.02^b^	3.49^a^	3.36^a^	0.148	0.016
Whole phase (days 1~63d)					
ADG (g/d)	41.89^a^	34.56^c^	35.99^b^	0.576	<0.001
ADFI (g/d)	101.84^a^	90.72^b^	94.90^b^	2.840	0.005
F:G	2.43^b^	2.63^a^	2.59^a^	0.066	0.023

a,b Mean values within a row with no common superscript differ significantly (*p*<0.05).

Ctr group, basal diet in control group; SNE group, basal diet+SNE (20-fold dose coccidiosis vaccine)+1ml of *Clostridium perfringens* (2×10^8^cfu/ml coinfection); BST group, basal diet (days 1–21)+1×10^9^ colony-forming units (cfu)/kg BS diet (days 22–63)+SNE. SEM, standard error of mean; BW, body weight; ADG, average daily gain; ADFI, average daily feed intake; and F:G, feed conversion ratio.

### Intestinal Lesion Scores

Intestinal lesion scores in the small intestines harvested on days 28 and 35 are presented in Table 4. Birds in all treatment groups had low lesion scores, indicating that the *C. perfringens* infection was subclinical. Higher lesion scores were observed in the SNE group in comparison to other groups, and a significant difference was observed on day 28 (*p*<0.05), which indicating that the infection was worsen in these birds. Interestingly, lesion scores in the BST group were not significantly different from those in the Ctr group (*p*>0.05).

**Table 4:** The jejunum histomorphology of broilers infected with *Clostridium perfringens*.

Items	Ctr	SNE	BST	SEM	**p**
Villus height (μm)	1437.22^a^	1047.27^b^	1128.68^b^	46.29	<0.001
Crypt depth (μm)	208.40^a^	188.85^a^	160.65^b^	9.29	<0.001
Villus/Crypt ratio	6.95^a^	5.55^b^	7.19^a^	0.43	0.001
Lesion score					
Day 28	0.50^b^	1.33^a^	0.83^ab^	0.22	0.002
Day 35	0.58	1.67	0.83	0.46	0.059

a,b Mean values within a row with no common superscript differ significantly (*p*<0.05).

Ctr group, basal diet in control group; SNE group, basal diet+SNE (20-fold dose coccidiosis vaccine)+1ml of *Clostridium perfringens* (2×10^8^cfu/ml coinfection); BST group, basal diet (days 1–21)+1×10^9^ colony-forming units (cfu)/kg BS diet (days 22–63)+SNE. SEM, standard error of mean. 0, no gross lesions, normal intestinal appearance; 1, thin walled or friable and few whitish plaques in the serosal surface (mild); 2, thin-walled, focal necrosis or ulceration, and small amounts of gas production (moderate); 3, thin-walled, large patches of necrosis, gas-filled intestine, and small flecks of blood (marked or severe); 4, severe extensive necrosis, marked haemorrhage, and excessive amounts of gas in the intestine (very severe).

### Histological Observation

**Figure 1:**
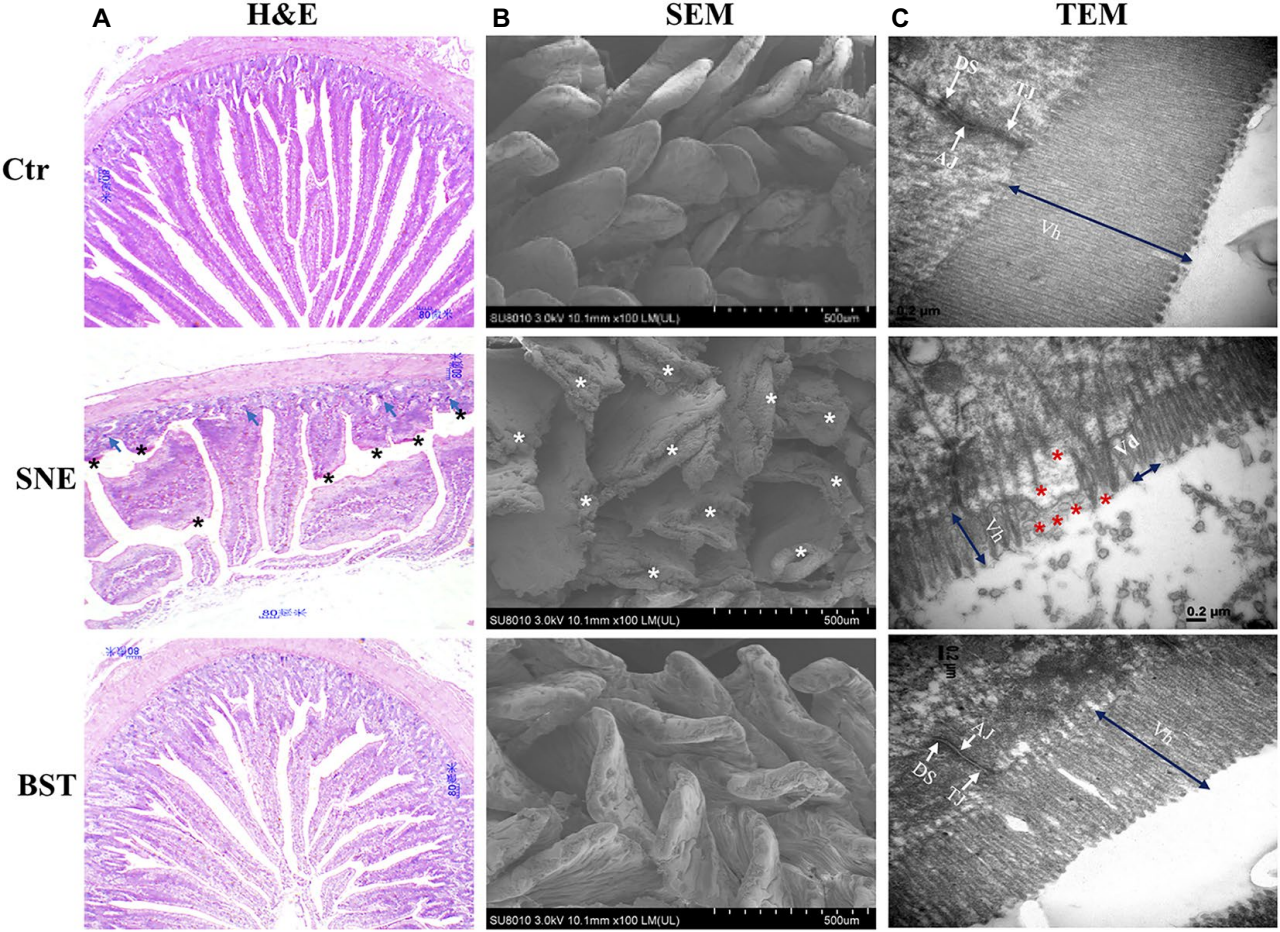
**(A)** Representative haematoxylin and eosin (H&E)-stained images (top, scale bars=80μm); **(B)** Scanning electron micrograph (bottom, scale bars=500μm); and **(C)** Transmission electron microscopy (bottom, scale bars=0.2μm) of jejunal mucosal surface of broilers orally treated with or without coccidia and *Clostridium perfringens* infection. Vh, Villus height; Vd, Villus density; TJ, tight junction; AJ, adherens junction; DS, desmosomes. Shows the pathological (asterisk). Numerous enterocytes show all the features of necrotic cell death (blue arrows). Ctr group, basal diet in control group; SNE group, basal diet+SNE (20-fold dose coccidiosis vaccine)+1ml of *Clostridium perfringens* (2×10^8^cfu/ml coinfection); BST group, basal diet (days 1–21)+1×10^9^ colony-forming units (cfu)/kg BS diet (days 22–63)+SNE.

H&E staining showed that the Ctr group exhibited an integrated structure of the jejunal mucosa, ordered intestinal villi, deep crypts, and a clear and complete gland structure (Figure 1A). However, tissues harvested from the SNE group showed that the structure of the jejunal mucosa was incomplete, villi were sparse and shorter, and the crypts were shallow with a sporadic distribution. The intestinal mucosal structure in chicks treated with BST was markedly improved, and the intestinal villi showed a longer and denser arrangement. Furthermore, BST significantly increased the villus height/crypt depth ratio compared with that observed in the SNE group, similar results were observed in the Ctr group (Table 4). SEM imaging showed that the Ctr group had complete jejunum villi structure, which formed full and closely arranged structures. In contrast, the jejunum villi structure in the SNE group was severely damaged, while those in the BST group showed an improvement (Figure 1B). These observations were also observed in by TEM imaging of the jejunum (Figure 1C).

### Expression of Genes Related to Intestinal Tight Junctions

**Figure 2:**
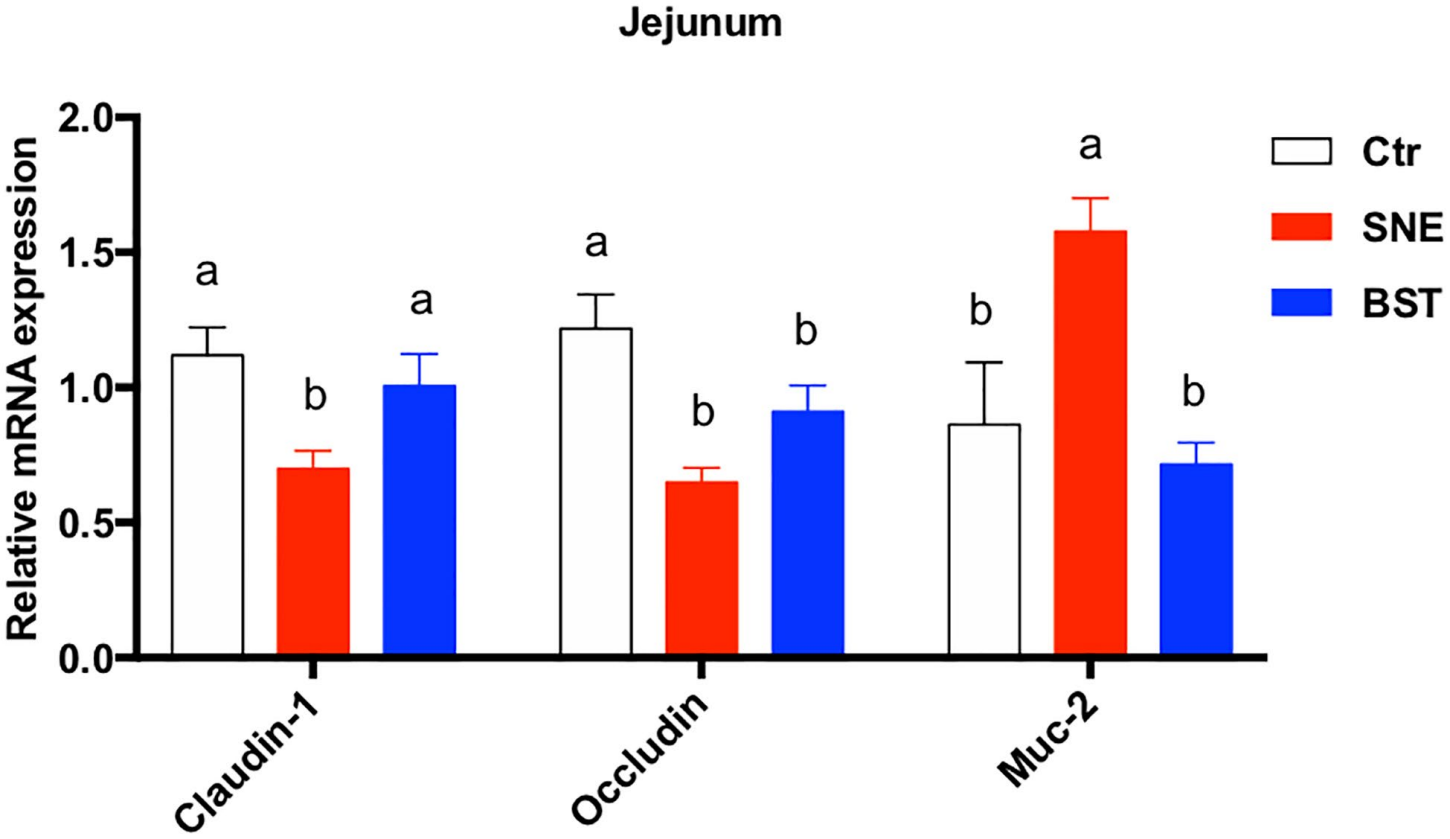
Changes in relative expression of *claudin-1*, *occluding*, and *muc-2* in the jejunum. Data are means±standard error (*n*=6). ^a,b^Mean values within a row with no common superscript differ significantly (*p*<0.05). Ctr group, basal diet in control group; SNE group, basal diet + SNE (20-fold dose coccidiosis vaccine)+1ml of *Clostridium perfringens* (2×10^8^cfu/ml coinfection); BST group, basal diet (days 1–21)+1×10^9^ colony-forming units (cfu)/kg BS diet (days 22–63)+SNE. *Muc-2*, *mucin 2*.

As shown in Figure 2, expression levels of claudin-1 and occludin transcripts in the jejunum were significantly decreased in the SNE group (*p*<0.05), compared to the Ctr group. However, no significant differences (*p*>0.05) in claudin-1 and occludin expression were observed between the BST and Ctr groups. Notably, the relative expression of Muc-2 in the SNE group was markedly upregulated (82.73% and 120.35%) compared to the Ctr and BST groups, respectively.

### Biochemical Indices in the Duodenum

Table 5 summarizes the effect of BS treatment on sucrase, amylase, and maltase enzymatic activity in the duodenal mucosa. Compared to the Ctr group, SNE significantly decreased (*p*<0.05) sucrase and maltase activity in the duodenum, whereas no significant difference was observed in BST group (*p*>0.05), except for maltase activity. In addition, no significant difference was observed for amylase activity in each treatment groups (*p*>0.05).

**Table 5:** Duodenal mucosa biochemistry parameters of SNE broilers treatment with *Bacillus subtilis* DSM29784 (mean±SEM, *n*=8 per treatment).

Items	Ctr	SNE	BST	SEM	*p*
Sucrase (U/mg prot)	62.97^a^	41.86^b^	46.51^ab^	9.019	0.070
Amylase (U/mg prot)	32.67	25.83	33.66	4.392	0.176
Maltase (U/mg prot)	104.79^a^	54.22^c^	90.52^b^	6.206	<0.001

a,b,c Mean values with unlike letters between different groups were significantly different (*p*<0.05).

Ctr group, basal diet in control group; SNE group, basal diet+SNE (20-fold dose coccidiosis vaccine)+1ml of *Clostridium perfringens* (2×10^8^cfu/ml coinfection); BST group, basal diet (days 1–21)+1×10^9^ colony-forming units (cfu)/kg BS diet (days 22–63)+SNE.

### Immune Response in Jejunal Mucosa

**Figure 3:**
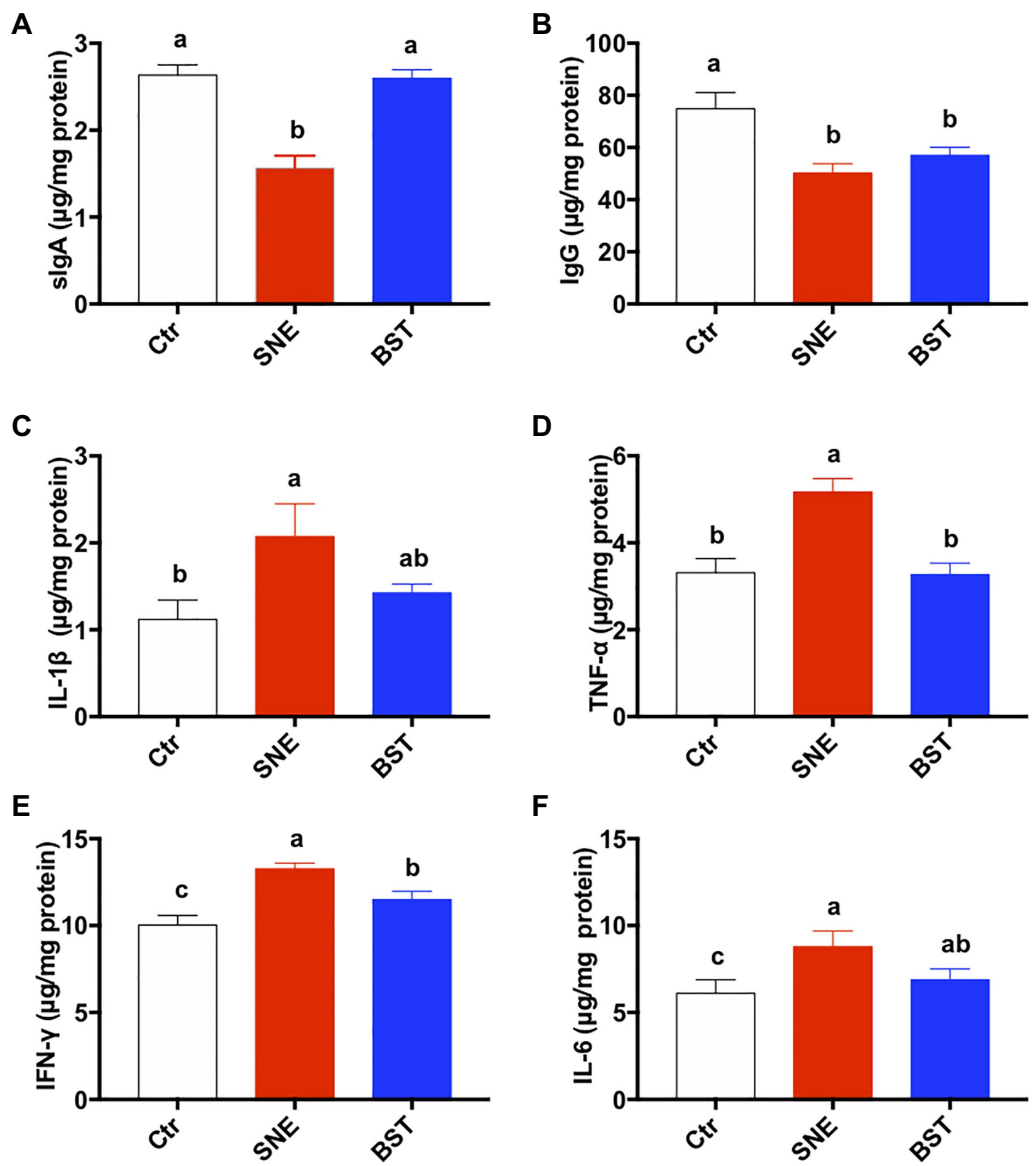
Cytokine levels in the jejunum. Bars with different letters significantly differ on the basis of Turkey’s multiple range tests (*p*<0.05). Data are presented as mean±SEM (*n*=8). sIgA, secretory IgA; TNF-α, tumor necrosis factor-alpha; IFN-γ, interferon-gamma. Ctr group, basal diet in control group; SNE group, basal diet+SNE (20-fold dose coccidiosis vaccine)+1ml of *Clostridium perfringens* (2×10^8^cfu/ml coinfection); BST group, basal diet (days 1–21)+1×10^9^ colony-forming units (cfu)/kg BS diet (days 22–63)+SNE.

Cytokine secretion in the jejunal mucosa is shown in Figure 3. The expression levels of sIgA and IgG were significantly lower in the SNE group than in the Ctr group (*p*<0.05), however no significant difference in sIgA secretion was noted between the BST and Ctr groups (*p*>0.05; Figures 3A,B). Furthermore, the secretion levels of IL-1β, TNF-α, INF-γ, and IL-6 were significantly upregulated in the SNE group, compared with those in the Ctr group. There were no significant differences between the BST and Ctr groups for these parameters except for the TNF-α and INF-γ (*p*>0.05; Figures 3C–F).

### Apoptosis-Related Proteins in the Jejunum

**Figure 4:**
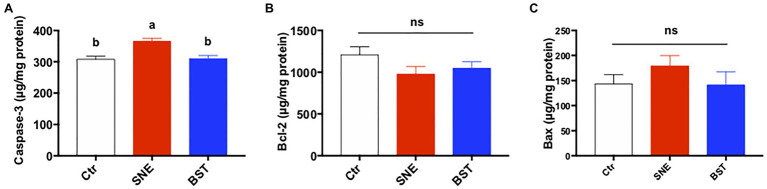
Levels of apoptosis-related proteins [caspase-3 **(A)**, bcl2 **(B)**, and bax **(C)**] in the jejunum. Bars with different letters significantly differ on the basis of Turkey’s multiple range tests (*p*<0.05). Data are presented as mean±SEM (*n*=8). Ctr group, basal diet in control group; SNE group, basal diet+SNE (20-fold dose coccidiosis vaccine)+1ml of *Clostridium perfringens* (2×10^8^cfu/ml coinfection); BST group, basal diet (days 1–21)+1×10^9^ colony-forming units (cfu)/kg BS diet (days 22–63)+SNE.

Figure 4 summarizes the levels of apoptosis-related proteins in the jejunum. Caspase-3 secretion in the jejunal mucosa of the SNE group was higher than both the Ctr and BST groups (*p*<0.05). In addition, no significant changes were observed in Bax and Bcl-2 levels among the three treatment groups (*p*>0.05).

### Microbial Community Structure of the Ceca

**Figure 5:**
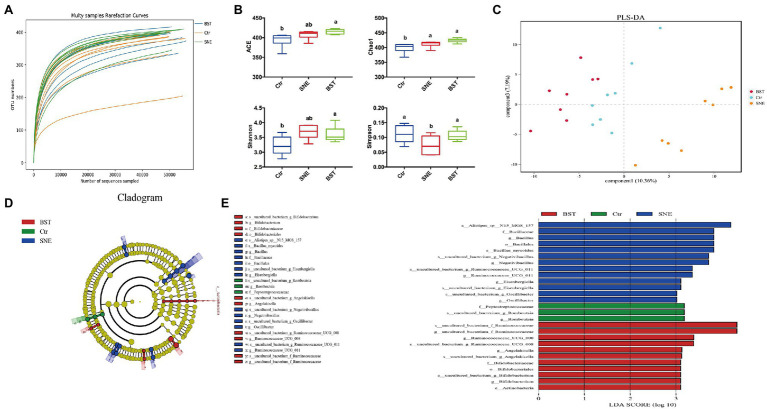
The caecal bacterial community of broilers fed with dietary *Bacillus subtilis* DSM 29784 supplementation among Ctr, SNE and BST treatments. **(A)** Rarefaction curve for total OTUs; **(B)** α-Diversity of gut microbial was analyzed among Ctr, SNE, and BST treatments by determination of principal dimension ACE, chao1, Shannon, and Simpson indices; **(C)** Partial Least Squares Discriminant Analysis of gut microbiota at the operational taxonomic unit (OTU) level; **(D)** Cladogram; and **(E)** LDA value distribution histogram. Bacterial taxa significantly differentiated among Ctr, SNE, and BST treatments identified by linear discriminant analysis coupled with effect size (LEfSe) using the default parameters. Bacterial taxa with LDA score>3 are selected as biomarker taxa (p: phylum level; c: class level; o: order level; f: family level; g: genus level). Ctr group, basal diet in control group; SNE group, basal diet+SNE (20-fold dose coccidiosis vaccine)+1ml of *Clostridium perfringens* (2×10^8^cfu/ml coinfection); BST group, basal diet (days 1–21)+1×10^9^ colony-forming units (cfu)/kg BS diet (days 22–63)+SNE.

Rarefaction curve analysis of OTUs in all samples approached a plateau (Figure 5A), indicating that the sampling depths were sufficient to capture the overall microbial diversity in all harvested samples. Figure 5B shows a significant difference (*p*<0.05) in the alpha diversity index (Simpson), while no differences (*p*>0.05) were observed in the ACE, Chao1, and Shannon indices between the SNE and BST groups. In addition, ACE, Chao1, and Shannon indices of broilers in the BST groups significantly increased (*p*<0.05) compared with those in the Ctr group.

Next, we applied PLS-DA to divide the samples into clusters, to identify differences between the harvested samples by establishing a model of the relationship between species abundance and sample category. As shown in Figure 5C, the Ctr, SNE, and BST groups were well separated, with 10.36 and 7.19% variation, explained by principal components 1 and 3, respectively.

A cladogram representative of the cecal microbiota and the predominant bacteria is shown in Figures 5D,E. The differences in taxa between the three groups are displayed. BST treatment significantly promoted the relative abundance of Ruminococcaceae (family), *Angelakisella* (genus), Bifidobacteriales (order), Bifidobacteriaceae (family), *Bifidobacterium* (genus), and Actinobacteria (phylum). SNE treatment markedly increased the relative abundance of Bacillaceae (family), *Bacillus* (genus), Bacillales (order), *Negativibacillus* (genus), *Eisenbergiella* (genus), and *Oscillibacter* (genus).

### Profiling of Cecal Metabolites

**Figure 6:**
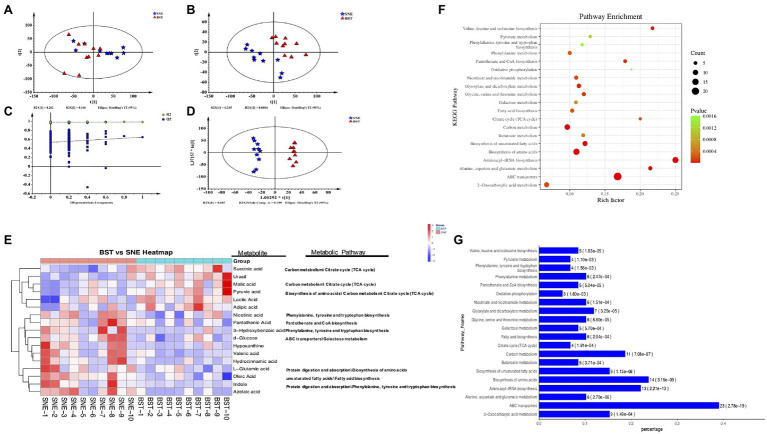
**(A)** Principal component analysis (PCA) score plots (R2X=0.534, Q2=0.252), **(B)** projections to latent structure-discriminant analysis (PLS-DA) score plots (R2X=0.52, R2Y=0.987, Q2=0.651), **(C)** permutation test of PLS-DA, and **(D)** orthogonal projections to latent structure-discriminant analysis (OPLS-DA) score plots (R2X=0.52, R2Y=0.987, Q2=0.507). Blue pentagon: SNE; red triangle: BST, green circle: R2; blue square: Q2. The dashed line represents the regression line for R2 and Q2; **(E)** Heatmap of metabolites in cecal contents between BST vs. SNE groups. The relative values for each of these metabolites are indicated by color intensity with the legend indicated at the right of the figure. Red and blue represent higher and reduced concentrations of metabolites in the BST and SNE groups; **(F)** Bubble (up) and **(G)** bar (down) charts of top 20 most KEGG enrichment analysis of the differential metabolites between BST vs. SNE groups. “Rich factor” means that the ratio of the DEMs number and the number of metabolites have been annotated in this pathway. The greater of the Rich factor, the greater the degree of enrichment (bubble chart); along *x*-axis values represents enrichment ratio. The horizontal axis represents the number of count, the corresponding value of *p* in brackets, and the vertical axis is pathway name (bar charts). Ctr group, basal diet in control group; SNE group, basal diet+SNE (20-fold dose coccidiosis vaccine) +1ml of *Clostridium perfringens* (2×10^8^cfu/ml coinfection); BST group, basal diet (days 1–21)+1×10^9^ colony-forming units (cfu)/kg BS diet (days 22–63)+SNE.

To identify differentially expressed metabolites in the cecum, we screened and compared the SNE and BST treatment groups by metabolomic profiling. Figure 6A shows the PCA score plot of the cecal samples from the two groups. There was a clear separation along the t [1] axis, indicating that the cecal contents were different in each group. To obtain an improved separation, and for better understanding of the variables responsible for the classification, a latent structure-discriminant analysis (PLS-DA) model was applied to screen markers for the metabolites responsible for maximum separation, by removing the systematic variation unrelated to grouping. As shown in Figure 6B, all the samples in the PLS-DA score plots were within the 95% Hotelling’s T^2^ ellipse. The R2Y value of this model, which represents the explained variance, was 0.962. Cross-validation indicated good predictive ability of this model with a relatively high Q2 value of 0.551. Similar to the PCA model, PLS-DA also exhibited a distinct separation between the two groups. Furthermore, a permutation test (Figure 6C) was applied to assess the robustness and predictive ability of the PLS-DA model, and the corresponding R2Y and Q2 intercept values were 0.52, and 0.651, respectively. This indicated that this model was satisfactorily effective. Similar results were obtained using OPLS-DA (Figure 6D).

### Differential Metabolite Analysis

We combined the multivariate OPLS-DA and VIP statistical analyses, and the *t*-test univariate statistical analysis value of *p* to screen for statistically differential metabolites between SNE and BST groups. A total of 86 metabolite biomarkers with similarity >700, VIP>1, and *p*<0.05 were filtered as shown in Supplementary Figure S1. These metabolites mainly comprise lipids, amino acids, carbohydrates, organic acids, and amines, such as lactic acid, fumaric acid, oleic acid, D-fructose, L-glutamic acid, and indole, which are involved in multiple biochemical processes. BST treatment group showed an upregulation of 35 metabolites, and a downregulation of 52 metabolites compared with SNE. Seventeen metabolites were selected and shown in a heat map (Figure 6E); the concentrations of succinic acid, uracil, malic acid, pyruvic acid, lactic acid, and adipic acid showed upregulation in BST group than the SNE group. On the contrary, the concentrations of azelaic acid, indole, oleic acid, L-glutamic acid, hydrocinnamic acid, valeric acid, hypoxanthine, D-glucose, 3-hydroxybenzoic acid, pantothenic acid, and nicotinic acid were higher in SNE. In short, there was a significantly differential expression pattern of metabolic profiles between chicks in the SNE and BST groups. The altered metabolome of the cecum during BS supplementation was associated with secondary metabolites involved in various amino acid and carbohydrate metabolic pathways. These metabolites included, d-glucose (galactose metabolism), l-glutamic acid (biosynthesis of amino acids), indole (protein digestion and absorption), succinic acid (tricarboxylic acid cycle), malic acid (carbon metabolism), and pyruvic acid (biosynthesis of amino acids).

### Analysis of Metabolic Pathways

We compared various cecal metabolites between the SNE and BST groups and identified the pathways in which they participate. Seventy-seven enriched pathways displayed comprehensive impact values. The top 20 KEGG enrichment analyses of the differential metabolites between the SNE and BST groups are shown in Figures 6F,G. These pathways included ABC transporters, aminoacyl-tRNA biosynthesis, amino acid biosynthesis, carbon metabolism, biosynthesis of unsaturated fatty acids, alanine, aspartate, and glutamate metabolism, valine, leucine, and isoleucine biosynthesis, glyoxylate and dicarboxylate metabolism, pantothenate and CoA biosynthesis, and glycine, serine, and threonine metabolism.

### Cecal Metabolome and Gut Microbiome Correlations

**Figure 7:**
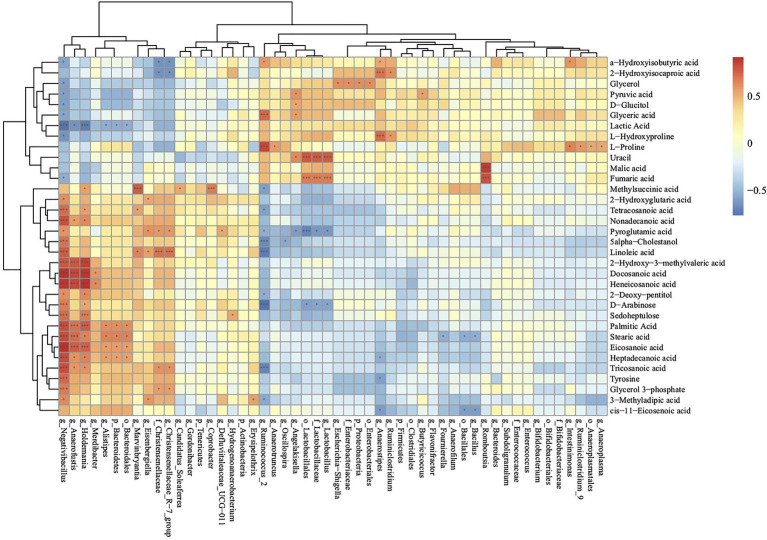
Correlations of metabolome and microbiota in cecal contents according to the Spearman correlation coefficient distribution (SNE vs. BS). *p>0.01 and *p*<0.05; ***p<0.01. Ctr group, basal diet in control group; SNE group, basal diet + SNE (20-fold dose coccidiosis vaccine) +1ml of *Clostridium perfringens* (2×10^8^cfu/ml coinfection); BST group, basal diet (days 1–21)+1×10^9^ colony-forming units (cfu)/kg BS diet (days 22–63)+SNE.

To further investigate the relationships between cecal metabolites and gut microbiota, Spearman correlation analysis was performed for cecal metabolites and microbiota in the BST an SNE groups (Figure 7). The relative abundance of Lactobacillales (order), Lactobacillaceae (family), and *Lactobacillus* (genus) were positively correlated with uracil and fumaric acid, and negatively correlated with pyroglutamic acid and D-arabinose. The relative abundance of *Negativibacillus* (genus), *Anaerofustis* (genus), *Holdemania* (genus), and *Alistipes* (genus) were positively correlated with palmitic acid, stearic acid, eicosanoic acid, heptadecanoic acid, and negatively correlated with lactic acid. In addition, the relative abundance of Ruminiclostridium was positively correlated with that of L-hydroxyproline and 2-hydroxyisocaproic acid.

## Discussion

SNE impairs growth performance, resulting in greater economic losses than the clinical form, as it can easily go unnoticed. Previous studies in have shown that probiotics have beneficial effects on the health of the host (Wang et al., 2017a). For example, the dietary probiotic *Bacillus* can effectively improve the growth performance and antioxidant capacity of animals and reduce the extent of intestinal lesions (Gobi et al., 2018; Jia et al., 2018; Musa et al., 2019). Our results show that the BST group, compared with the SNE group, showed beneficial effects in BW during the final phases of the experiment, and a higher ADG over the duration of the experiment. Moreover, previous findings are consistent with our current study. For instance, Jayaraman et al. (2017) reported that dietary supplementation with *B. subtilis* could be used as an alternative to antibiotic growth promoters in poultry farming, as it has exhibited good growth performance in birds.

Intestinal health and recovery are evaluated based on villi length and crypt depth. A higher ratio of villus length: crypt depth illustrates that the villus is longer and has a mature and active functional epithelium, accompanied by a shallow crypt and constant cell renewal (Jayaraman et al., 2013). Increasing villi length provides a greater intestinal surface area and improves nutrient absorption as well as the growth and production of broilers (Salim et al., 2013). In contrast, shorter villi and deeper crypts may lead to decreased disease resistance, poorer growth performance, and reduced nutrient absorption (Song et al., 2014; Mohammadagheri et al., 2016). Thus, changes in intestinal conditions can affect the nutrient absorption of birds. In this study, BS treatment effectively improved jejunum morphology, including villi length and the villi length: crypt depth ratio in the broilers. The same results were reported when broilers were administered *B. subtilis*, which improved the health of the intestine by enhancing the villus length, width and area, and the crypt depth in the small intestine (Bai et al., 2018). The above-mentioned findings illustrate that improved intestinal morphology development may contribute to the preventive of SNE.

Barrier function can be influenced by several luminal and systemic factors, leading to plasma protein leakage and watery diarrhea (Camilleri, 2019). Tight junctions provide paracellular permeability, which is integral to the intestinal mucosal barrier (Tugizov, 2021) and contain unique proteins, such as Claudin-1, occludin, and Muc-2 (Wang et al., 2018). Intestinal epithelial cells secrete mucus onto their surface, which is a major protective barrier against bacterial infection. However, intestinal mucus is an important nutritional source for CP, as it allows these bacteria to form part of the local microflora on the mucosal surface (Ficko-Blean et al., 2012). Here, we found that BST treatment significantly decreased the relative expressions of Muc-2 compared to the SNE group. Reduced mucus secretion may reduce CP localization, thus providing a preventive effect against SNE.

Previous studies have considered as an immune-metabolic perspective to identify the role of the inflammatory response mediating some diseases, such as obesity, diabetes, and other metabolic disorders. For instance, excessive fat deposition can lead to an innate immune inflammatory response (Pothakam et al., 2021). Host–pathogen interactions in NE are complex and involve different components of the host immune system (Oh and Lillehoj, 2016). Cytokines are effective molecules for the transmission of information between immune cells. The composition of cytokines in the infection microenvironment determines the properties of the immune response (Fasina and Lillehoj, 2019). We observed a greater expression of pro-inflammatory cytokines in the SNE than other two groups, while excessive inflammation can lead to tissue damage (David and Lopez-Vales, 2021). Fasina and Lillehoj (2019) showed that CP infection could induce intestinal inflammation in broilers, which may be mediated by Threshold (Th) 2 and Th17 cells. sIgA antibody, as the first line of antigen-specific immune defense, protects the mucosal surface from environmental pathogens and antigens by combining with bacterial antigens (Tiboni et al., 2021). We also found that BST treatment upregulated the expression levels of sIgA in the jejunum. This is consistent with our previous results showing that probiotics increase the expression of sIgA and triggers the production of cytokines (Wang et al., 2015; Wu et al., 2019). The above-mentioned findings illustrate that BST treatment may help to relieve the inflammatory response and protect the intestine against pathogenic bacterial infection.

NE can alter the composition and quantity of gastrointestinal microbiota, which plays a pivotal role in the digestive tract of animals (Stanley et al., 2014; Azad et al., 2018). SNE is a type of dysbacteriosis, an imbalance of the normal flora in the proximal small intestine, caused by a variety of opportunistic pathogens (Palliyeguru and Rose, 2014). Previous studies have shown that probiotics can actively regulate the composition of the intestinal microbiota (Kristensen et al., 2016; Hu et al., 2017). *Ruminococcus* forms part of the natural flora in the intestinal tract of chickens. In human studies, some *Ruminococcus* flora help cells to absorb sugars and may contribute to weight gain (Correa et al., 2021), which explains why the BST chickens gained weight over the duration of the experiment, compared to the other treatment groups. The present study showed that the relative abundance of *Ruminococcaceae* in the BST group was higher than that in the other groups. Ahiwe et al. (2019) showed a decrease in the diversity of *Lactobacillus* and *Bifidobacterium* in the cecum of the CP challenged broilers. Notably, an increase in the population of *Bifidobacterium* spp. in the cecum was observed in the BST group, suggesting that BS treatment reduced the effect of NE on the composition and quantity of gastrointestinal microbiota. Khan et al. (2016) demonstrated that when *B. subtilis* was used as a probiotic, microbial balance in the chicken gut could be improved by immune stimulation and competitive exclusion. Similarly, *B. subtilis* can reduce the relative abundance of CPs in the intestine by changing the microflora (Bortoluzzi et al., 2019).

The greatest bacterial density in broilers is found in the cecum (Rehman et al., 2007). A complex microbial community in the intestine plays a key role in nutrition and health (Yang and Zhao, 2021). For example, probiotics and their metabolites can promote a symbiotic balance of microorganisms in the gut of the broilers (Perez-Chabela et al., 2020). Metabolomics provide novel insights into the dynamics of metabolites in the gut of broilers. For instance, different feed additives can directly affect the types of metabolites in the intestinal tract (Dittoe et al., 2018). Here, the higher concentrations of metabolites identified in BST group primarily include organic acids, amino acids, and alcohols. Various organic acids (such as lactic, succinic, nicotinic, acetic, propionic, and malic) are produced during intestinal fermentation and form barriers in the digestive tract, thereby preventing the colonization and reproduction of pathogenic bacteria. This enhances peristalsis and digestive enzyme secretion in the intestinal tract, thus promoting feed digestion, and improved nutrient absorption (Sun et al., 2020).

In chickens, most energy comes from glucose through the digestion of starch and long-chain fatty acids (Jozefiak et al., 2004). The decreased levels of D-glucose and sedoheptulose in the ceca of broilers in the BST treatment group might have been attributed to the increased adsorption of this carbohydrate. In addition, certain carbohydrates are metabolized by bacteria in the gut to produce different types of organic acids, together with volatile fatty acids produced from carbohydrate fermentation, such as branched chain fatty acids and lactic acid, which play an important role in the gastrointestinal tract of birds, for instance, by inhibiting the growth of certain pathogenic bacteria. This is achieved by fatty acids penetrating the bacterial cell membrane, leading to the dissociation of charged anions and protons, a decrease in intracellular bacterial pH, and inhibition of essential metabolic reactions, ultimately reducing bacterial replication (Cherrington et al., 1991; Youssef et al., 2020). Our results showed higher levels of lactic, succinic, α-hydroxyisobutyric, and malic acid in the BST group than in SNE broilers, which may explain the better health status of broilers in the BST group. Similar to our results, previous studies have reported a reduction in NE signs using butyric acid and sodium lauroyl lactylate in the diets of NE challenged birds (Biggs and Parsons, 2008; Huyghebaert et al., 2011).

Amino acids play a vital role in regulating the nutritional metabolism in broilers. Proteins consumed by broiler chickens are degraded into free amino acids by proteases and peptidases in the gastrointestinal tract and absorbed through the intestinal wall. Different types of amino acids were identified in BST broilers as compared to SNE broilers, with the composition observed in BST broilers more conducive to growth. Ammonia is generated by proteolytic bacteria, such as *Clostridium* spp., *Enterococcus* spp., and *Bacteroides* spp., and is released with potentially toxic metabolites such as biogenic amines, phenols, and indole (Macfarlane et al., 1992). Ammonia is easily absorbed by the intestine into the blood of birds and causes enterocytes toxicity (Marleen et al., 2003). Our data demonstrated that both the levels of indole and monoethanolamine were markedly increased in the cecal contents of SNE broilers compared to the BST group, suggesting that BS supplementation can alleviate the toxic side effects of CP infection. In addition, KEGG pathway annotation showed that several metabolic pathways were altered in the BS-treated broilers compared to the SNE group. This enrichment mainly involved lipid and amino acid metabolic pathways, such as protein digestion and absorption, biosynthesis of unsaturated fatty acids, and biosynthesis of amino acids.

Microorganisms and their associated metabolites are a natural component of the developmental process and have a significant, yet underexplored, impact on the immune system (Wandro et al., 2018). In addition to the direct interaction with the immune system, the microbiota also interacts indirectly through the production of metabolites, which can be absorbed by the immune and epithelial cells (Wikoff et al., 2009; Dodd et al., 2017). In our study, the relative abundance of beneficial bacteria was positively correlated with a marked increase in metabolites in the BST group, and negatively correlated with the increase in metabolites in the SNE group. This suggests that the changes in cecal metabolites might have originated from the intestinal microbiota following co-infection of the coccidiosis vaccine and *C. perfringens*. The strong correlation between intestinal microflora and metabolites observed in the SNE broilers suggests that significant changes may cause an imbalance in the host immune response, which ultimately decline the growth performance of broilers.

**Figure 8:**
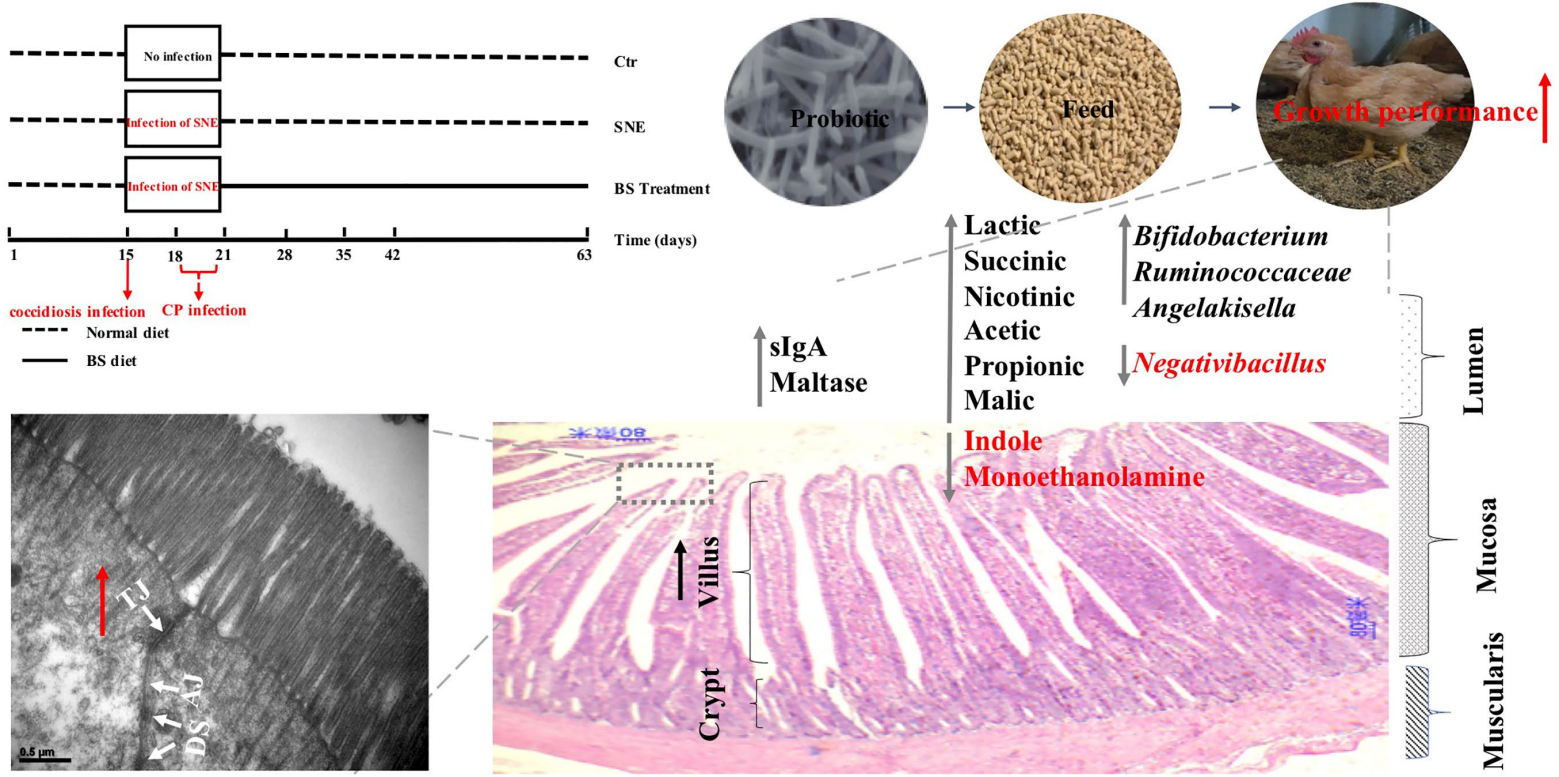
Graphical summary of the effect of probiotic *Bacillus subtilis* DSM29784 (BS) exerts a protective effect against subclinical necrotic enteritis in broilers. BS reduces the risk of declined growth performance in chickens with SNE by enhancing exoenzyme secretion, intestinal barrier functions, and promoting the presence of beneficial commensal microorganisms and their metabolites in the intestine. SNE, subclinical enteritis; sIgA, secretory immunoglobulin A; CP, *Clostridium perfringens*; TJ, tight junction; AJ, adherens junction; DS, desmosomes. Ctr group, basal diet in control group; SNE group, basal diet+SNE (20-fold dose coccidiosis vaccine) +1ml of *Clostridium perfringens* (2×10^8^cfu/ml coinfection); BST group, basal diet (days 1–21)+1×10^9^ colony-forming units (cfu)/kg BS diet (days 22–63)+SNE.

In conclusion, results from the current study indicated that treatment with BS reduces the risk of declined BW in chickens with SNE by enhancing exoenzyme secretion, intestinal barrier functions, immunity, decreasing cell apoptosis, and promoting the presence of beneficial commensal microorganisms and their metabolites in the intestine (Figure 8). We intend to further investigate whether BS supplementation throughout the experimental period is a successful alternative for controlling clostridial infection in broilers.

### Data Availability Statement

The datasets presented in this study can be found in online repositories. The names of the repository/repositories and accession number(s) can be found in the article/Supplementary Material.

## Ethics Statement

The animal study was reviewed and approved by the Zhejiang University Institutional Animal Care and Use Committee (permission number: ZJU2019-480-12).

## Author Contributions

XZ, YW, KW, and JY designed the experiments. SX, YW, and YX performed animal husbandry. YW and KW analyzed 16S rRNA data. YW and YX did the animal experiments. YW authored the final article. All authors contributed to the article and approved the submitted version.

## Funding

This work was supported by a Zhejiang Province Key R&D Program of China (project: 2018C02035, Hangzhou, China), China Agriculture Research System of MOF and MARA (project: CARS-41, Beijing, China), Agricultural Science and Technology Cooperation Program of Zhejiang Province (project: 2021SNLF024, Hangzhou, China), and Collaborative Extension Plan of Major Agricultural Technologies in Zhejiang Province (project: 2021XTTGXM04).

## Conflict of Interest

The authors declare that the research was conducted in the absence of any commercial or financial relationships that could be construed as a potential conflict of interest.

## Publisher’s Note

All claims expressed in this article are solely those of the authors and do not necessarily represent those of their affiliated organizations, or those of the publisher, the editors and the reviewers. Any product that may be evaluated in this article, or claim that may be made by its manufacturer, is not guaranteed or endorsed by the publisher.

## Footnotes

^1^ https://www.ncbi.nlm.nih.gov/sra

^2^ http://qiime.org/

^3^ http://huttenhower.sph.harvard.edu/lefse/

## Abbreviations

ABC: ATP-binding cassette
ADFI: Average daily feed intake
ADG: Average daily gain
BCA: Bicinchoninic acid
BS: *Bacillus subtilis* DSM 29784
BST: BS treatments groups
BW: Body weight
CAT: Catalase
cfu: Colony-forming units
CP: *Clostridium perfringens*
ELISA: Enzyme-linked immunosorbent assay
F:G: Feed: gain ratio
GSH-Px: Glutathione peroxidase
H&E: Haematoxylin and eosin
HRP: Horse radish peroxidase
IFN-γ: Interferon-gamma
IgG: Immunoglobulin G
IL: Interleukin
KEGG: Kyoto Encyclopedia of Genes and Genomes
LDA: Linear discriminant analysis
MDA: Malondialdehyde
MUC2: Mucin 2
NE: Necrotic enteritis
PBS: Phosphate-buffered saline
PBST: Phosphate-Buffered Saline Tween
PCA: Principal component analysis
PCR: Polymerase chain reaction
SEM: Scanning electron microscopy
sIgA: Secretory immunoglobulin A
SNE: Subclinical necrotic enteritis
SOD: Superoxide dismutase
SPSS: Statistical Product and Service Solutions
TEM: Transmission electron microscopy
TMB: Tetramethy1 benzidine
TNF-α: Tumor necrosis factor α
VIP: Variable importance in projection
