# Supplementary material for: Bacillus subtilis DSM29784 Alleviates Negative Effects on Growth Performance in Broilers by Improving the Intestinal Health Under Necrotic Enteritis Challenge
Source: Front Microbiol. 2021 Sep 16;12:723187. doi: 10.3389/fmicb.2021.723187 (PMC8481782; doi:10.3389/fmicb.2021.723187)
Supplement: Supplementary file 1 [file Data_Sheet_1.docx]

Supplementary Material

**Figure S1.** Heatmap of metabolites in cecal contents between SNE and BST groups. The relative values for each of these metabolites is indicated by color intensity with the legend indicated at the right of the figure. Red and blue represent higher and reduced concentrations of metabolites in the BST and SNE groups. Ctr group, basal diet in control group; SNE group, basal diet + SNE (20-fold dose coccidiosis vaccine + 1 ml of *C. perfringens* (2 × 10^8^ cfu/ml coinfection); BST group, basal diet [days 1-21]+ 1×10^9^ colony-forming units (cfu)/kg BS diet [days 22-63] + SNE.
